# Analysis of DNA Origami Nanostructures Using Capillary Electrophoresis

**DOI:** 10.1021/acs.analchem.3c03641

**Published:** 2023-12-13

**Authors:** Janan Hui, Jacob M. Majikes, Kathryn R. Riley

**Affiliations:** † Department of Chemistry and Biochemistry, Swarthmore College, Swarthmore, Pennsylvania 19081, United States; ‡ Physical Measurement Laboratory, National Institute of Standards and Technology (NIST), Gaithersburg, Maryland 20899, United States

## Abstract

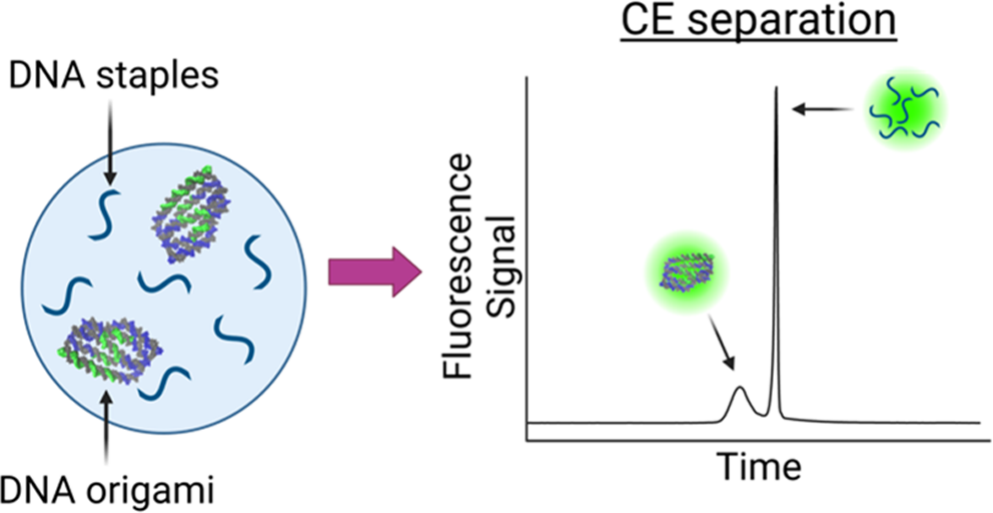

DNA origami nanostructures are engineered nanomaterials (ENMs) that possess significant customizability, biocompatibility, and tunable structural and functional properties, making them potentially useful materials in fields, such as medicine, biocomputing, biomedical engineering, and measurement science. Despite the potential of DNA origami as a functional nanomaterial, a major barrier to its applicability is the difficulty associated with obtaining pure, well-folded structures. Therefore, rapid methods of analysis to ensure purity are needed to support the rapid development of this class of nanomaterials. Here, we present the development of capillary electrophoresis (CE) as an analytical tool for DNA origami. CE was investigated under both capillary zone electrophoresis (CZE) and capillary transient isotachophoresis (*ct*ITP) modes. Optimization of both systems yielded baseline resolved separations of folded DNA origami nanostructures from excess staple strands. The *ct*ITP separation mode demonstrated superior performance in terms of peak resolution (*R*_s_ = 2.05 ± 0.3), peak efficiency (*N* = 12,200 ± 230), and peak symmetry (*A*_s_ = 1.29 ± 0.032). The SYBR family dyes (Gold, Green I, and Green II) were investigated as highly efficient, noncovalent fluorophores for on-column labeling of DNA origami and detection using laser-induced fluorescence. Finally, *ct*ITP analysis conditions were also applied to DNA origami nanostructures with different shapes and for the differentiation of DNA origami aggregates.

## Introduction

A one-pot, bottom-up assembly of DNA nanostructures developed by Rothemund in 2006, so-called “DNA origami”, is widely used today for the synthesis of complex DNA nanostructures.^1^ Bionanotechnology has widespread applications in medicine, bioelectronics, biocatalysis, and agriculture.^2^ For example, DNA nanostructures have enormous potential in applications, such as drug delivery, biosensing, protein functionalization, scaffolding, programmable circuitry, enzyme cascades, and more.^3^

During DNA origami synthesis, short “staple” oligonucleotides (typically 15–60 nucleotides, nt, long) fold a long “scaffold” (900–8000 nt long) of single-stranded DNA into complex structures stabilized by thousands of base pair interactions. These staple strands are typically added in excess, so the removal of these construction materials from the folded DNA origami is essential to avoid interference in further experiments and applications.^4^ DNA origami’s primary application is as a “smart glue”, capable of arranging a wide array of proteins, molecules, and nanoparticles. The DNA origami community therefore relies heavily on workflows that include iterative addition, characterization, and purification. Unfortunately, the characterization of folded structures is challenging due to the small size of DNA origami (approximately 7.5 × 10^–18^ g or 4.5 MDa), so new tools capable of detecting the origami and distinguishing its features are needed to advance the field.

Characterization of formed DNA origami is conventionally done through various imaging techniques, such as atomic force microscopy (AFM), electron microscopy, fluorescence microscopy, and super-resolution microscopy. AFM is the most widely used technique for 2D origami due to its cost efficiency, high sensitivity, and ability to resolve structural features or measure material properties.^5−8^ Transmission electron microscopy (TEM), fluorescence microscopy, and super-resolution microscopy are able to provide 3-dimensional structural analysis, image fast and dynamic assembly processes, and probe single-molecule interactions, respectively.^9,10^ Yet, the broad applicability of these techniques is hindered because they require invasive and tedious sample preparation with different conditions for each unique origami sample, have high background signals, and are expensive.^5^ Other characterization techniques like gel electrophoresis or DNA melting curves may be used to provide information on structural and thermal stability of the origami; however, as bulk techniques, they are not particularly sensitive to sample heterogeneity.^11^

Overall, current characterization techniques are not able to be widely applied to different DNA origami structures without the need for extensive optimization and suffer from poor resolution of heterogeneous samples (e.g., mixtures of properly folded and misfolded structures). For these reasons, evaluating new analysis techniques is a source of interest in the DNA origami community.^12^ Capillary electrophoresis (CE) is an electrokinetic separation method that is ideally suited for the analysis of DNA.^13−16^ Using CE, analytes are separated based on differences in their charge and size, and in the case of analytes with significant surface area (e.g., a folded DNA origami structure), analytes are also separated based on frictional drag forces. Further, CE instruments may be coupled with UV–vis absorbance, laser-induced fluorescence, electrochemiluminescence, or mass spectrometric detectors to enable online characterization.

Herein, we present the development of capillary electrophoresis (CE) as an analytical tool for DNA origami. CE was investigated and optimized under both capillary zone electrophoresis (CZE) and capillary transient isotachophoresis (*ct*ITP) modes. Both systems yielded baseline resolved separations of folded DNA origami nanostructures from excess staple strands. The *ct*ITP separation mode demonstrated superior performance in terms of peak amplitude, peak efficiency, and peak symmetry. The SYBR family dyes (Gold, Green I, and Green II) were investigated as highly efficient, noncovalent fluorophores for on-column labeling of the DNA origami and detection using laser-induced fluorescence. Finally, *ct*ITP analysis conditions were applied to DNA origami nanostructures with different shapes and for the differentiation of DNA origami aggregates, demonstrating the potential broad applicability for DNA origami characterization.

## Experimental Section

Certain commercial equipment, instruments, or materials are identified in this paper to specify the experimental procedure adequately. Such identifications are not intended to imply recommendation or endorsement by the National Institute of Standards and Technology, nor it is intended to imply that the materials or equipment identified are necessarily the best available for the purpose.

### Chemicals

DNA origami, including a tripod,^17^ notched rectangle (herein, NR), rope, and pillar morphologies, were annealed from M13MP18 single-stranded DNA bacteriophage folded by staple strands sourced from Integrated DNA Technologies. Staple strands were added in excess (≈500 nmol·L^–1^) to scaffold (≈50 nmol·L^–1^) so that the scaffold was fully consumed to yield origami with a final concentration of ≈50 nmol·L^–1^.

There are 7249 nucleotides per M13MP18 ssDNA scaffold molecule. Some scaffold nts and staple nts are left as ssDNA to control the flexibility or prevent aggregation of the structures. The staple pools contain 7284, 2859, 6611, and 7188 nts spread across 224, 199, 176, and 208 oligomers for the NR, rope, pillar, and tripod staple pools, respectively. Origami were therefore comprised of approximately 7000 base pairs of dsDNA with a few hundred nts of ssDNA. Lists of the staple strand sequences needed to fold the M13MP18 scaffold into each origami structure are provided in the Supporting Information. All DNA samples (staples, scaffolds, and folded origami) were stored at 4 °C between analyses.

Magnesium chloride (≥99.0%), magnesium acetate tetrahydrate (≥99.0%), ethylenediaminetetraacetic acid (anhydrous, ≥99.0%), Trizma base (≥99.5%), glycine (≥99.0%), 3-(*N*-morpholino)propanesulfonic acid (MOPS), 4-(2-hydroxyethyl)piperazine-1-ethanesulfonic acid (HEPES), hydrochloric acid (≥99.0%), and sodium hydroxide were purchased from MilliporeSigma (Burlington, MA). SYBR Gold, SYBR Green I, and SYBR Green II nucleic acid gel stains were purchased as 10,000× concentrates, 5 mmol·L^–1^, from Invitrogen (Thermo Fisher Scientific, Carlsbad, CA).

### Sample and Buffer Preparation

For CZE optimization experiments, a background electrolyte (BGE) containing varying concentrations of Trizma base (10–60 mmol·L^–1^), magnesium acetate (2.5–12.5 mmol·L^–1^), and EDTA (1 mmol·L^–1^) was prepared in Millipore water (18.2 mΩ·cm at 25 °C). This buffer is henceforth referred to as TAE buffer. SYBR intercalating dyes were added to the buffer and the dye: the BGE volume ratio was optimized between 1:10,000 and 1:100,000. The buffer was adjusted to the appropriate pH (between 7 and 9) by using 1 mol·L^–1^ HCl. The BGE was stored in the dark at 4 °C for up to 1 week. The final selected buffer for CZE analysis was 60 mmol L^–1^ Tris, 5 mmol·L^–1^ magnesium acetate, and 1 mmol·L^–1^ EDTA, pH 8.0 with SYBR Green I added at a 1:25,000 dye/buffer ratio.

For *ct*ITP experiments, two buffers were prepared; one served as the sample buffer and contained the leading ion, Cl^–^, while the other served as the separation buffer and contained the terminating ion, glycine (Gly). The sample buffer was used to dilute the DNA samples, and the separation buffer was used to fill the capillary. The final selected CZE buffer was used as the sample buffer for *ct*ITP (60 mmol·L^–1^ Tris, 5 mM magnesium acetate, and 1 mmol·L^–1^ EDTA, pH 8.0). The *ct*ITP separation buffer was selected by varying the concentrations of Trizma base (10–60 mmol·L^–1^) and glycine (0.1–1 mol·L^–1^). Again, SYBR intercalating dyes were added to the separation buffer and the dye: the BGE volume ratio was optimized between 1:10,000 and 1:100,000. The final chosen buffer contained 40 mmol·L^–1^ Tris and 500 mmol·L^–1^ glycine (pH 8.5) with SYBR Green I added at a 1:100,000 dye/buffer ratio. The *ct*ITP sample and separation buffers were stored in the dark at 4 °C for up to 1 week.

### Capillary Electrophoresis of DNA Origami Structures

CE-LIF experiments were performed using a SCIEX P/ACE MDQ Plus capillary electrophoresis system equipped with a 488 nm Ar-ion laser. Separations were carried out using a fused-silica capillary of customizable length (varied from 40.2 to 60.2 cm total length) and diameter (50 or 75 μm). All buffers and solutions used to flush the capillary were filtered through a 0.20 μm nylon syringe filter before use. Each day, the capillary was flushed successively for 10 min with Millipore H_2_O, 10 min with 0.1 mol·L^–1^ NaOH, 10 min with Millipore H_2_O, and 20 min with either the CZE BGE or the *ct*ITP separation buffer. A blank electropherogram was recorded by injecting either the BGE (for CZE analyses) or the sample buffer (for *ct*ITP analyses) and was used for baseline normalization of the fluorescence signal to zero.

Unless otherwise stated, all DNA samples (staples, scaffold, and/or origami) were diluted to a concentration of 0.5 nmol·L^–1^ in either the BGE (for CZE analyses) or in the sample buffer (for *ct*ITP analyses). During origami annealing, staples are present in a 10× excess to the scaffold and are consumed at a 1:1 ratio with the scaffold in conversion to origami. As such, the ratio of nts between the analyte band in which both the scaffold and origami migrate and the peak associated with staples should be between 1:10 and 2:9. Samples were injected hydrodynamically for 2 s at 34.5 kPa, or 5 psi, (19 nL injection volume) and the separation was carried out at 20 kV. Between each injection, the capillary was flushed with Millipore H_2_O for 1 min and either the CZE BGE or the *ct*ITP separation buffer for 2 min. All samples were analyzed in triplicate to demonstrate the reproducibility of the electropherogram profile.

32 Karat Software and OriginPro were used for data visualization and analysis. Analytical figures of merit for the separations were calculated using eqs S1–S3.

### Spin Filtration of DNA Origami Structures

To facilitate proof-of-concept for the analysis of DNA origami by CE, migration times of the various origami structures were verified by first purifying the origami from the staples and scaffold using spin filtration and analyzing the purified structures using CE. Amicon Ultra-0.5 centrifugal filter units (molecular weight cutoff = 3 kg·mol^–1^ or 3 kDa) were used to carry out spin filtration (MilliporeSigma). Samples were pipetted onto the spin filter membrane, and an Eppendorf MiniSpin Plus microcentrifuge was operated at 11,000*g* for 5 min. Samples were centrifuged 10 times in succession. Then, samples were retrieved by inverting the spin filter in a new Eppendorf tube, adding the appropriate volume of buffer, and centrifuging for 10 s at 11,000*g*.

### Fluorescence Characterization of SYBR Intercalating Dyes

A PTi fluorescence spectrometer was used for data collection. The cuvette chamber was thermostated at 25 °C and fluorescence spectra were recorded from 500 to 600 nm using an excitation wavelength of 488 nm, a scan rate of 2 nm·s^–1^, and excitation and emission slit widths of 4.0 nm. Although the excitation maxima for each dye are unique, 488 nm was used as the excitation wavelength for the analysis of all dyes to mirror the wavelength of the Ar-ion laser used in CE experiments. Excitation and emission spectra were recorded for the dye diluted 1:10,000 in 60 mmol·L^–1^ Tris, 5 mmol·L^–1^ magnesium acetate, and 1 mmol·L^–1^ EDTA buffer (pH 8.0) either in the absence or presence of 0.5 nmol·L^–1^ DNA staples. Then, emission scans were collected for the staples, scaffold, and each DNA origami structure for all three SYBR dyes. DNA origami were spin-filtered to remove excess staples prior to fluorescence analysis and diluted to a final concentration of 0.5 nmol·L^–1^. All samples were prepared and analyzed in triplicate.

### Microscopy of DNA Origami Structures

AFM images were collected for each of the formed origami. A buffer (typically the TAE buffer used for the origami synthesis) was loaded onto freshly cleaved mica, and the origami was pipetted onto the mica surface. The sample was scanned in liquid tapping mode beginning with an amplitude set point of 200 mV using a three-sided AFM probe with a tip radius of approximately 8 nm (biolever mini BL-AC40TS).

TEM images were collected from dry samples deposited on carbon-coated TEM grids that were plasma-ashed to provide a charged surface and were either imaged directly or with a negative stain (uranyl acetate).^17^

## Results and Discussion

**Figure 1:**
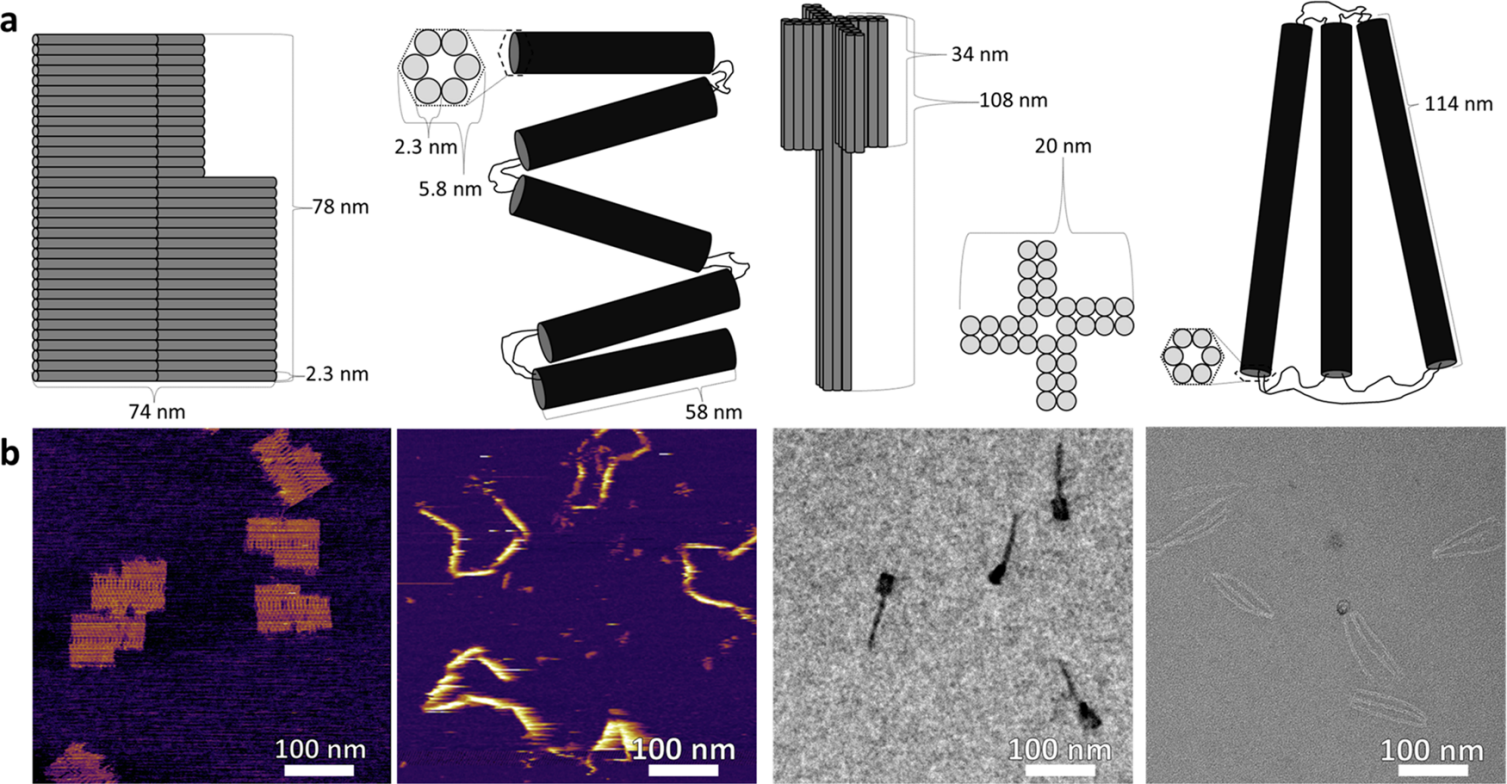
(a) Schematics and estimated physical dimensions for (from left to right) the NR, rope, pillar, and tripod DNA origami structures with expected, rather than measured, dimensions. (b) Images verifying the formation of (from left to right) the NR (AFM, liquid tapping), rope (AFM, liquid tapping), pillar (TEM, unstained), and tripod (TEM, negative stained).

The DNA origami used in this study include ones previously reported, the NR and tripod,^17,18^ and two new designs, a pillar and rope. These origami were chosen for the variety of their 3D shape. The NR is planar, with a single rigid direction parallel to the helices and a flexible direction perpendicular to the helices. The tripod and rope are 3D structures comprised of loosely connected bundles, which are, in turn, comprised of 6 interconnected helices and are differentiated by the bundle lengths and the topology of their flexible connections. The pillar is a single 3D pillar that should be much more rigid than either the tripod or rope. All four designs have approximately the same final molecular weight, ≈4.5 × 10^6^ g·mol^–1^ (4.5 MDa), and are depicted in Figure 1.

**Figure 2:**
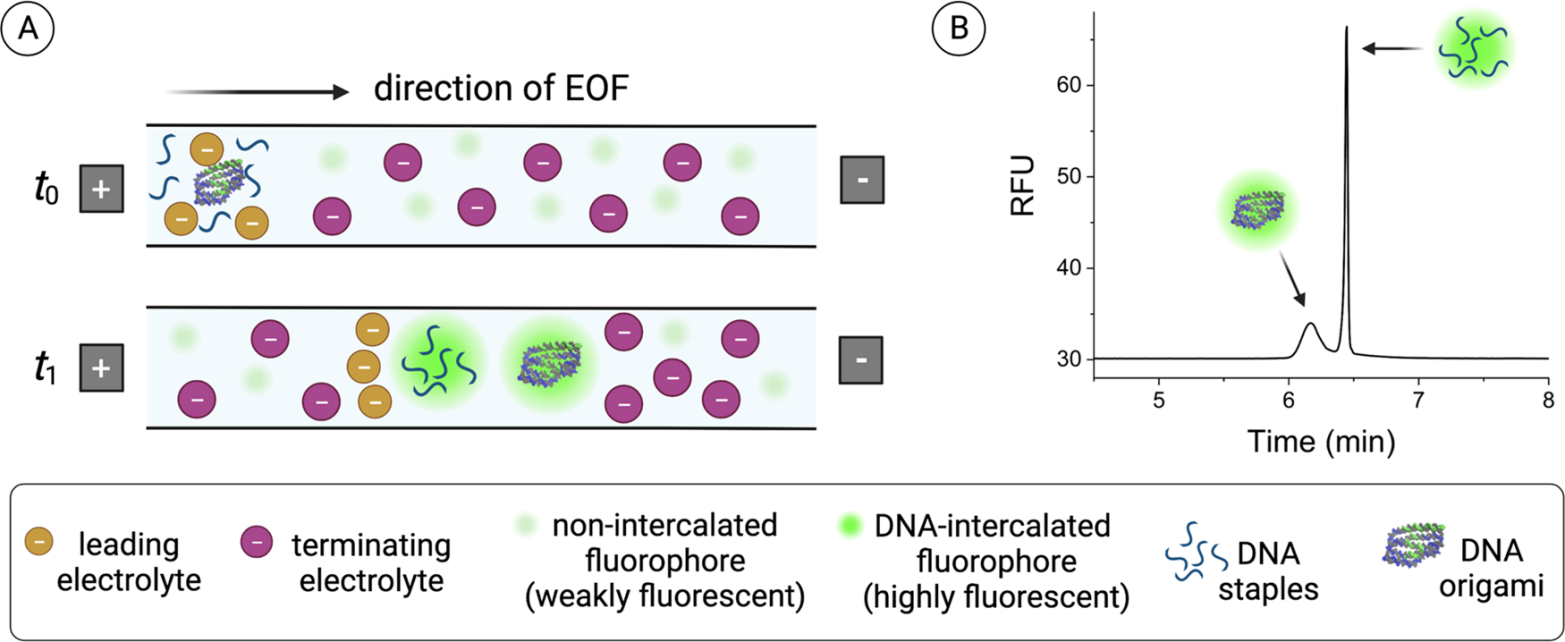
Schematic of CE-based analysis of DNA origami nanostructures. (A) Schematic of the CE-based separation of the NR DNA origami from excess staple strands. Capillary transient isotachophoresis (*ct*ITP) was used to focus the analyte peaks into narrow bands. DNA samples were labeled on-column with a noncovalent fluorophore that is weakly fluorescent on its own and exhibits intense fluorescence upon DNA intercalation. (B) Representative electropherogram showing the high resolution and peak efficiencies of separated analyte bands, where RFU stands for relative fluorescence units.

The development of CE for the analysis of DNA origami structures is illustrated in Figure 2. Briefly, NR origami structures were chosen as an example nanostructure to optimize the separation of DNA origami from staple strands. Using our optimized conditions, the NR and excess staple strands are injected into the capillary and separated using *ct*ITP (Figure 2A). DNA analytes are labeled on-column using a SYBR family intercalating dye, and separated analyte bands are detected using laser-induced fluorescence detection (Figure 2B). We evaluated both the CZE and *ct*ITP separation modes. CZE uses a continuous buffer system with a single background electrolyte (BGE) that fills the capillary and in which samples are prepared. Analytes are separated by their unique electrophoretic mobilities, which depend on the charge and size of the analyte. For analytes with sufficient surface area like the formed DNA origami, frictional drag forces play a larger role, and in this case, facilitate separation of the origami from excess DNA staples. However, despite the ease of sample preparation, CZE can suffer from significant band broadening. To reduce band broadening, focusing techniques like *ct*ITP may be used, whereby the sample is stacked between the leading and terminating electrolyte with greater or less mobility than the sample, respectively.^19−22^ During the stacking period, longitudinal diffusion of the sample is minimized.

Development of the CE analysis method included systematic optimization of the following experimental parameters: choice of fluorescent dye (SYBR Gold, SYBR Green I, or SYBR Green II) and its concentration; buffer composition, concentration, and pH; and CE conditions, including capillary dimensions, sample injection conditions, and separation voltage. The final selected conditions were chosen based on reproducibility of analyte migration times, reduction of band broadening, and improvements in peak resolution. A mixture of staple strands and the M13 scaffold (concentrations ≈0.5 nmol·L^–1^) was used as a model system to optimize the separation conditions since they were readily obtained at larger volumes (several milliliters) and higher concentrations (500 nmol·L^–1^) than the formed DNA origami (several microliters, 50 nmol·L^–1^). The mixture of staple strands and the scaffold had an electrophoretic profile similar to that of the unpurified NR origami and was deemed as an appropriate proxy for conditions to isolate the NR (Figure S1). During origami annealing, the staple pools are introduced to the scaffold in a 10× excess and react in a 1:1 stoichiometry with the scaffold, and it is generally assumed that the scaffold is completely converted to origami. While intercalating dyes typically have lower quantum yields when bound to ssDNA than dsDNA, the relative excess of staple strands results in a more intense fluorescence peak (Figure 2B).^23^

Following the optimization of separation conditions for the analysis of the NR, we also investigated the broad application of *ct*ITP by analyzing other DNA origami structures (tripod, rope, and pillar) and detecting aggregated structures.

### Noncovalent Fluorescent Labeling of DNA Origami Using SYBR Family Dyes

**Figure 3:**
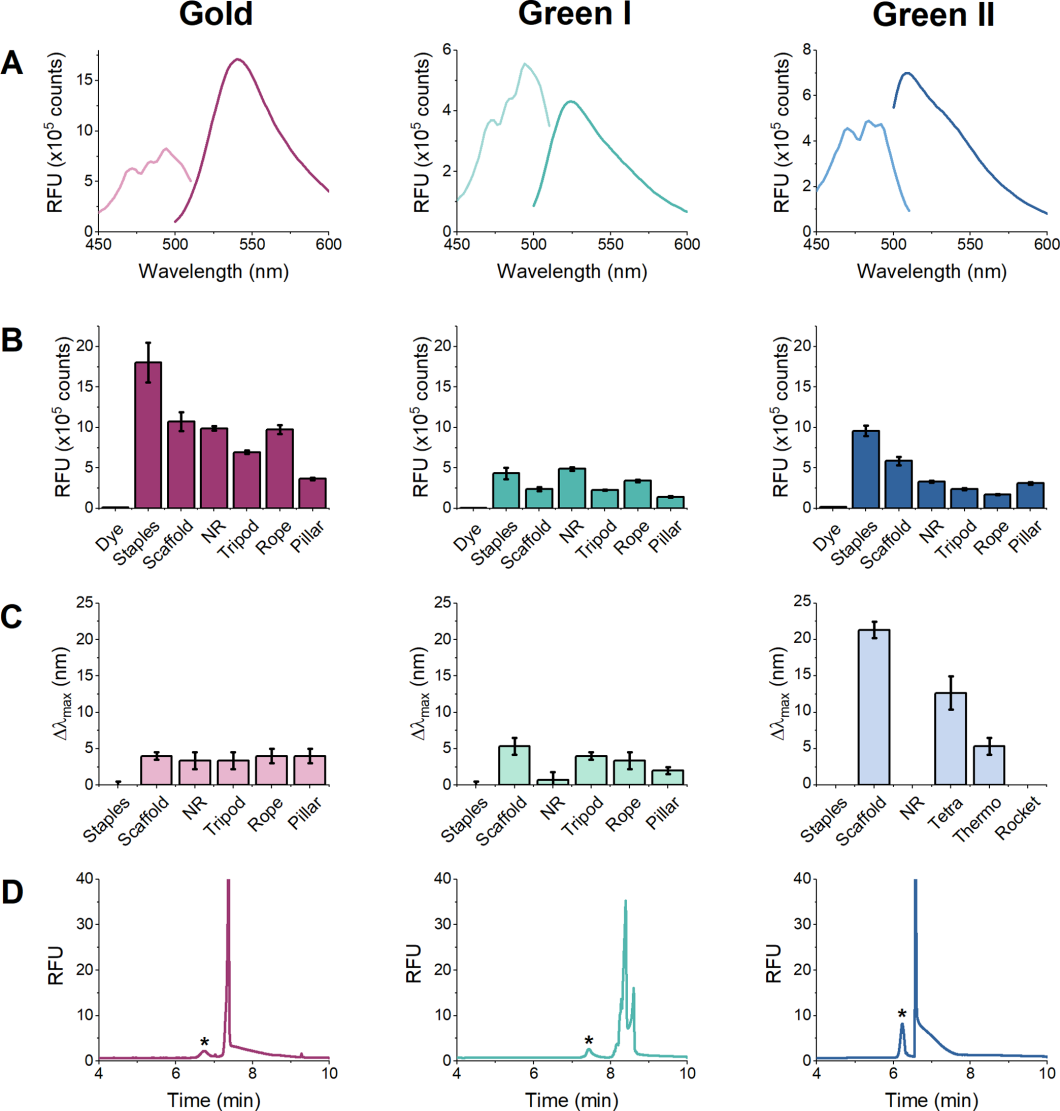
(A) Excitation and emission spectra of SYBR intercalating dyes incubated with DNA origami staple strands. (B) Fluorescence emission signals of 0.5 nmol·L^–1^ DNA origami (staples (for NR), scaffold, NR, tripod, rope, and pillar) with SYBR intercalating dyes. (C) Change in the λ_max_ of the SYBR dye emission band upon DNA intercalation. (D) *ct*ITP electropherograms showing the separation of the NR DNA origami (marked with an asterisk, *) from excess staple strands. Fluorescence experiments (A–C) were carried out using a 1:10,000 dilution of the dye and samples were prepared in 60 mmol·L^–1^ Tris, 5 mmol·L^–1^ magnesium acetate, and 1 mmol·L^–1^ EDTA buffer (pH 8.0). *ct*ITP experiments were carried out using a 40 mmol·L^–1^ Tris and 500 mmol·L^–1^ Gly separation buffer (pH 8.0) with the indicated dye added at a 1:100,000 ratio. Samples were prepared in the same buffer as used for fluorescence experiments, and a 15 kV separation voltage was applied (≈250 V·cm^–1^). Uncertainty bars represent the standard deviation of at least three measurements.

First, we explored the labeling efficiency of the SYBR family dyes for the staple strands, scaffold, and purified samples of each origami structure using fluorescence spectroscopy (Figure 3). Probing the affinity of the dyes for different origami structures can yield important information on the dynamics of intercalating dyes used for applications such as biosensing, and in the context of this work, it helped in selection of a dye for CE optimization. The excitation and emission spectra of SYBR Gold, SYBR Green I, and SYBR Green II were recorded in the presence of DNA origami staple strands using a 488 nm excitation wavelength and a 540 nm emission wavelength (Figure 3A). When compared to the emission of the dyes alone, the fluorescence of the dyes in the presence of ssDNA staples was enhanced by factors of 140, 93, and 54 for SYBR Gold, Green I, and Green II, respectively. This is consistent with the enhancement observed in previous studies, where SYBR Gold was shown to be more sensitive than SYBR Green I or II for detection of both single-stranded and double-stranded DNA.^24^

The fluorescence signals of different DNA origami samples also demonstrate the diversity of binding affinity based on the double-stranded and single-stranded characteristics of each structure (Figure 3B). For instance, the flat, essentially two-dimensional NR structures showed consistently greater fluorescence compared to the more morphologically complex tripod that has more double-stranded character on the double-helical sides. Further, depending on the DNA sample and the specific dye analyzed, the λ_em_ of the dye red-shifted relative to the staple strands (Figure 3A,C). A redshift or blueshift in the emission maximum is commonly observed for the SYBR family dyes and other cyanine dyes and is attributed to a change in the polarity of the local environment of the dye upon DNA intercalation.^25−28^ Overall, SYBR Gold exhibited superior performance across all DNA structures and was used for the initial optimization of CE separation conditions described below.

Following fluorescence spectroscopy analysis, an experiment was conducted to compare the effectiveness of on-column and precolumn labeling of DNA origami in CE. Precolumn labeling involves adding the fluorophore to the sample prior to injection, which is typically less efficient since the dye can dissociate from the DNA as it migrates along the capillary leaving a very small fraction of the analyte labeled by the time the analyte reaches the detection window. In comparison, by filling the capillary with the dye (on-column labeling) even as the dye dissociates from the DNA, new dye molecules along the length of the capillary can quickly re-establish the DNA–dye complex. This enables a larger proportion of the DNA sample to be labeled when it reaches the detection window compared to precolumn labeling. For a sample of DNA staples, a 300-fold enhancement was observed when using on-column compared to precolumn labeling (Figure S2). For a sample mixture of NR origami and DNA staples labeled precolumn, the migration peak of the NR was below the limit of detection and only a small migration peak was observed for the staples (Figure S2, blue trace, inset). In comparison, using on-column labeling, a distinct peak was observed for the NR origami and a very intense peak was observed for the staples (Figure S2, green trace). Thus, all CE analyses employed on-column labeling.

### Capillary Zone Electrophoresis (CZE)

**Figure 4:**
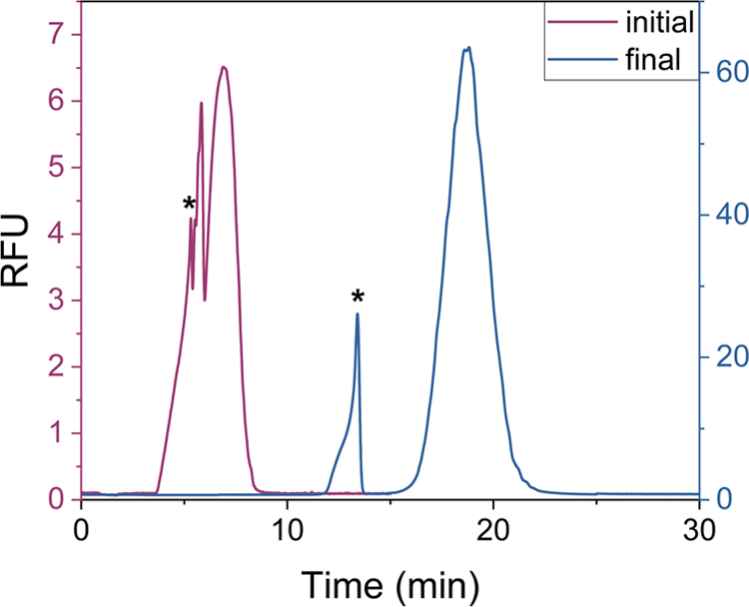
Representative electropherograms demonstrating optimization of CZE conditions for the separation of the NR (marked with an asterisk, *) and excess staple strands. The “initial” separation conditions utilized a 30.0 cm capillary (*L*_effective_) and a separation buffer of 40 mmol·L^–1^ Tris, 12.5 mmol·L^–1^ magnesium acetate, and 1.0 mmol·L^–1^ EDTA (pH 7.5) with SYBR Green I added to the buffer in a 1:100,000 ratio. The “final” separation conditions utilized a 50.0 cm capillary (L_effective_) and a separation buffer of 60 mmol·L^–1^ Tris, 5.0 mmol·L^–1^ magnesium acetate, and 1.0 mmol·L^–1^ EDTA (pH 8.0) with SYBR Green I added to the buffer in a 1:25,000 ratio. A 20 kV separation voltage was applied in both cases (≈500 and ≈330 V·cm^–1^ for the initial and final, respectively).

Initial CZE conditions, including the buffer composition (40 mmol·L^–1^ Tris, 12.5 mmol·L^–1^ magnesium acetate, 1 mmol·L^–1^ EDTA, pH 7.5), choice of SYBR dye (SYBR Gold), and SYBR dye concentration (1:100,000 dilution), were adapted from literature demonstrating the application of CZE for the analysis of DNA (Table S1).^14,22,29^ The electropherogram obtained from these initial separation conditions showed a broad band of unresolved peaks from ≈4 to 8 min (Figure 4, pink trace). The peak labeled with an asterisk (*) was determined to be the NR origami through comparison to electropherograms of individual injections of staple strands and origami purified through spin filtration. The unresolved third peak positioned between the NR peak and the excess staple peak may be attributed to heterogeneity in the solute band due to poorly formed NR or unfavorable dispersive phenomena and interactions with the capillary during the long migration times.^30^

The concentration of SYBR Gold was optimized first due to the low fluorescence intensity of the NR band. As the SYBR dye concentration was increased (from a 1:100,000 dye/buffer ratio to a 1:10,000 ratio), the reproducibility of the peak migration time, area, and width generally improved (Table S2). In addition, the migration time of DNA staples decreased, and the peak efficiency increased as the SYBR Gold concentration was altered (Figure S3), suggesting that the addition of SYBR Gold to the separation buffer has a slight impact on electroosmotic flow (EOF). Beyond a 1:25,000 dye/buffer ratio, no additional gains in detection sensitivity or peak reproducibility were observed, so the 1:25,000 dye/buffer ratio was chosen for proceeding experiments.

Next, the buffer conditions, including pH and concentrations of Mg^2+^ and Tris, were optimized. Overall, modifying the buffer pH and Tris concentration had the most significant impact on the reproducibility of the analyte peak (Table S2) and its migration time and intensity (Figures S4–S6). Other zwitterionic buffers were also explored, including MOPS and HEPES. Despite the improved peak efficiency afforded by MOPS (Figure S7), data for these buffers were not as reproducible as the Tris buffer (Table S2).

Using the selected BGE conditions (60 mmol·L^–1^ Tris, 5.0 mmol·L^–1^ Mg(CH_3_COO)_2_, 1 mmol·L^–1^ EDTA, pH 8.0, 1:25,000 SYBR Gold/buffer ratio), a reproducible, Gaussian peak for the staples sample was obtained. These conditions were then applied to mixtures of the staples and NR scaffold to optimize instrument separation parameters (e.g., voltage, injection, and capillary dimensions). Increasing the capillary length had the most significant impact on the separation; while peak migration times and band broadening substantially increased, the resolution of staples and scaffolds was also substantially improved (Figure S8). The separation voltage (10, 15, and 20 kV) and injection pressure (13.8–34.5 kPa, or 2–5 psi, for 5 s) were also varied for improved separation performance. The applied voltage of 20 kV gave an ideal balance between reducing band broadening while maintaining an intense fluorescence signal. At injection pressures beyond 27.6 kPa, or 4 psi, the separation between the scaffold and staple peak was no longer observable.

The final selected CZE separation conditions are listed in Table S1. These conditions were applied for the analysis of an unpurified NR and yielded improved detection sensitivity and baseline resolution of the NR origami structure from the staple strands (Figure 4, blue trace). Despite the reproducibility of this separation, the optimized CZE system suffered from long analysis times, broad analyte bands, and poor peak symmetry. Thus, we next pursued the optimization of *ct*ITP as a means to focus analyte bands and achieve a higher-quality separation between the NR and staples.

### Capillary Transient Isotachophoresis (*ct*ITP)

Fundamental relationships observed in the CZE system were applied to the *ct*ITP separation mode in a similar optimization effort. The final selected BGE from CZE was used as the sample buffer for *ct*ITP, where the chloride anion from pH adjustment of the Tris buffer with HCl served as the leading ion (mobility greater than that of the analyte). The initial choice of terminating electrolyte was derived from the literature^22,31^ and was composed of 31 mmol·L^–1^ Tris −500 mmol·L^–1^ Gly, where Gly served as the terminating ion (mobility less than the analyte). Using *ct*ITP, a significant enhancement in peak intensity and reduction in band broadening was observed (Figure S9, blue trace), but improvements to the terminating electrolyte could further the distinction between the NR and excess staple strands.

Due to the increased sensitivity of *ct*ITP, the SYBR Gold concentration was decreased by a factor of 4 compared to CZE to avoid saturating the detector (final dilution of 1:100,000 dye/buffer). Then, injection parameters were tuned within the same ranges as those for CZE. A substantial improvement in the separation was observed by decreasing both the separation voltage and injection pressure (Figure S9 and Table S3). Next, the concentration of Tris was varied in 10 mmol·L^–1^ increments from 10 to 50 mmol·L^–1^ and the concentration of glycine from 100 mmol·L^–1^ to 1 mol·L^–1^ in 100 mmol L^–1^ increments. Ultimately, a Tris concentration of 40 mmol·L^–1^ and a Gly concentration of 500 mmol·L^–1^ afforded the best resolution of the NR from excess staple strands (Figures S10 and S11 and Table S3).

As CE optimization progressed from using CZE to *ct*ITP, the choice of SYBR dye was re-evaluated. Since *ct*ITP offered excellent detection sensitivity, the slight improvement in fluorescent intensity offered by SYBR Gold seemed less important, and we sought to identify the best dye for the separation and detection of the analytes. Using *ct*ITP separation conditions, SYBR Green II yielded the most intense DNA origami peak (marked with an asterisk in Figure 3D), while SYBR Green I afforded the greatest baseline resolution. Ultimately, SYBR Green I was chosen as the most effective dye for the separation, as its improved resolution enabled the greater detection of DNA origami from staple strands.

### Comparison of CZE and *ct*ITP for the Analysis of DNA Origami

**Figure 5:**
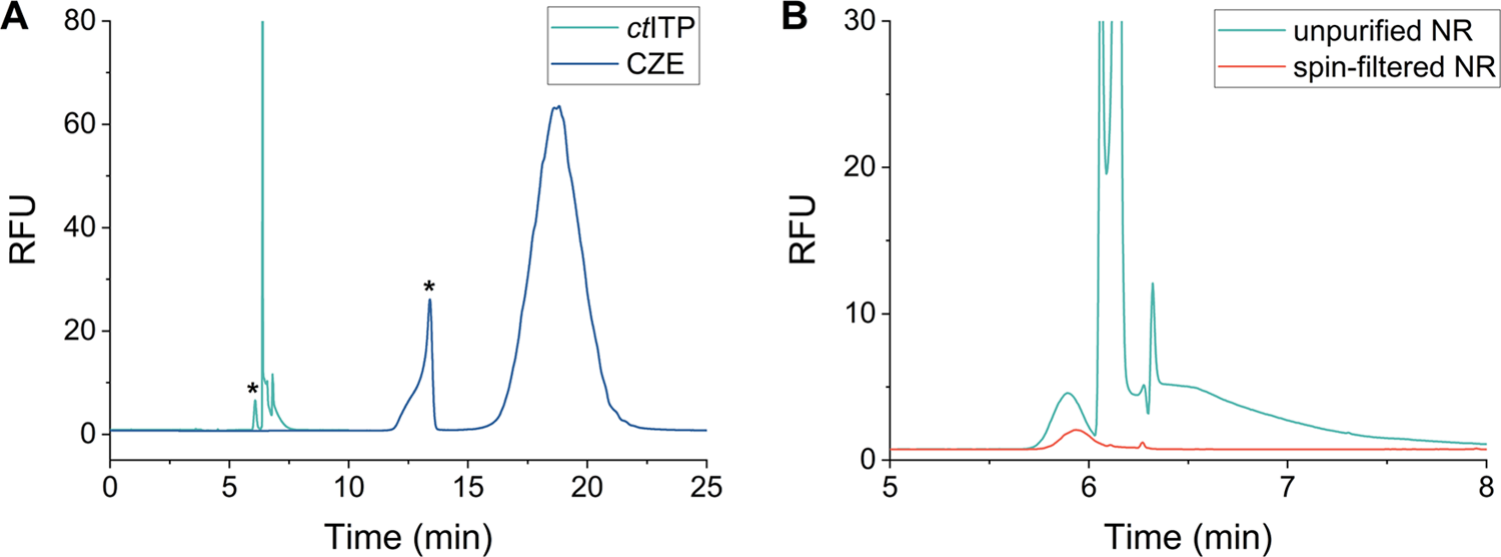
(A) Overlaid electropherograms demonstrating the optimized separation of the NR from excess staple strands using either CZE or *ct*ITP. CZE separation conditions were as indicated in Figure 4 (“final”) and *ct*ITP separation conditions were as indicated in Figure 3. NR origami peaks are labeled with an asterisk (*). (B) Overlaid *ct*ITP electropherograms compare the unpurified NR containing the folded NR and excess staple strands with the NR purified using spin filtration and containing the folded NR only.

The final selected CZE and *ct*ITP separation conditions are reported in Table S4. Application of these conditions to the unpurified NR sample yielded baseline resolution of the NR from the staple strands in both cases (Figure 5A). A direct comparison between the optimized CZE system and *ct*ITP shows that the separation is achieved under significantly less time (within 24 min to within 8 min) and with significantly decreased band broadening. While the fluorescence intensity of the CZE condition may appear to be superior to the *ct*ITP condition, SYBR Green I had to be diluted by a factor of 4 when employing *ct*ITP to avoid saturating the detector. Overall, *ct*ITP offered a superior separation regarding several analytical figures of merit (Table 1). Compared to CZE, *ct*ITP had a better resolution factor (2.05 compared to 1.57), substantially sharper peaks, and a 3-fold decrease in analysis time. The number of theoretical plates, *N*, was calculated to quantify the peak efficiency for each separation mode. *ct*ITP had improved peak efficiency with an approximately 10-fold increase in the value of *N*. Finally, the peak asymmetry factor, *A*_s_, was calculated to show the improvement in the Gaussian nature of the NR peak, with *A*_s_ > 1, indicating peak tailing, and *A*_s_ < 1, indicating peak fronting. For the CZE system, *A*_s_ was calculated as 0.213, suggesting severe fronting, as depicted in the electropherogram. Meanwhile, for the *ct*ITP system, *A*_s_ was determined to be 1.29, indicating only slight tailing. As such, it was concluded that the *ct*ITP system was superior in performance compared to that of the CZE system.

**Table 1:** Analytical Figures of Merit for CZE and *ct*ITP Separation of NR and Staple Strands^a^

system	analysis time (min)	*R*_s_	*N*	*A*_s_
CZE	25	1.57 ± 0.14	1010 ± 140	0.21 ± 0.08
*ct*ITP	10	2.05 ± 0.30	12,160 ± 230	1.29 ± 0.03

a Analytical figures of merit were calculated using eqs S1–S3. Uncertainty values represent the standard deviation of three replicate measurements.

As a final confirmation of the identity of the separated peaks, origami samples that were purified by using spin filtration were injected under optimized *ct*ITP conditions to confirm the hypothesized migration order. The resulting electropherogram shows the spin-filtered NR migration peak aligning with the NR migration peak from the unfiltered sample, whereas the staple peak is no longer visible (Figure 5B). This is consistent with gel electrophoresis of the unfiltered and spin-filtered origami (Figure S12), in the latter of which the staple band was not visible.

### Differential Migration of Unique DNA Origami Nanostructures and Aggregates

**Figure 6:**
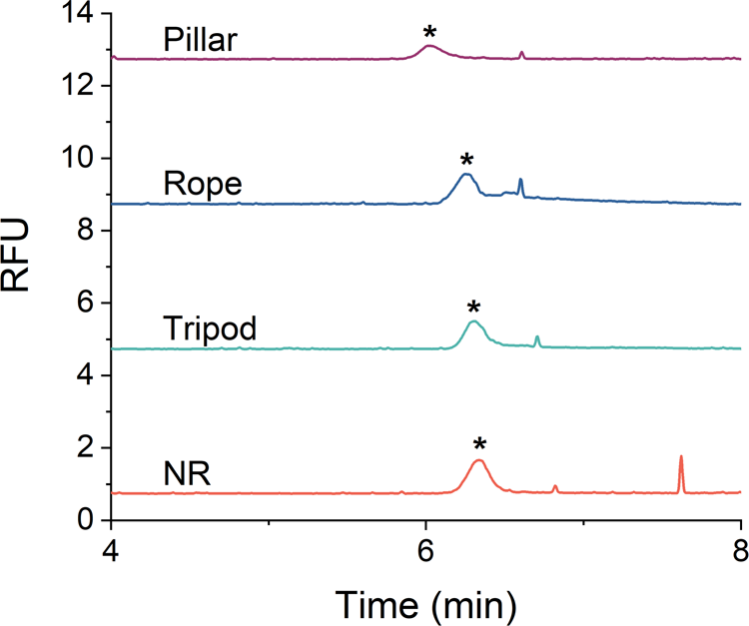
*ct*ITP electropherograms of DNA origami structures purified using spin filtration. Electropherograms are vertically offset for clarity, and the separation conditions were as indicated in Figure 3.

As a further demonstration of the utility of CE-LIF for the analysis of DNA origami nanostructures, we used the optimized *ct*ITP conditions to analyze spin-filtered samples of other DNA nanostructures (tripod, rope, pillar) and to characterize DNA origami aggregates. Analysis of the tripod, rope, and pillar yielded a peak profile similar to that of the NR (Figure 6). The pillar had the least intense peak, which is likely due to less efficient fluorescent labeling, consistent with our observations from the fluorescence spectroscopy study (Figure 3B). In addition, the different origami had unique migration times, with the order of migration observed as pillar, rope, tripod, and NR. These results suggest that *ct*ITP has the capability to resolve differences in the origami shape, presumably due to differences in their frictional drag during migration. While our separation conditions were selected for the separation of origami from excess staple strands, *ct*ITP conditions could be optimized for other intended applications, such as the separation of different origami structures or the separation of correctly folded structures from misfolded structures.

Interestingly, we noticed that the electropherogram profile for NR origami samples changed after several days of storage in the *ct*ITP sample buffer. In particular, we observed a new peak at an earlier migration time that was unresolved from the primary folded origami peak. The NR sample was incubated at varying lengths of time to investigate changes that occur in the electropherogram profile. It was determined that after a 3-day incubation period in the sample buffer, a new peak appeared that became more intense with longer incubation time (Figure S13). We also noticed a shift in the migration of the entire profile, which may be attributed to the aging of the separation buffer or the origami sample. The formation of new peaks could be explained by the NR design that lacks additional staple strands at the sides to “cap” the structure. Without these sides, base pairing can easily occur between two or more origami, leading to the formation of dimers or larger aggregates (Figure S14A). Aggregation of the NR sample would be consistent with the observation of a new peak at an earlier migration time, as the larger aggregated structure would be expected to migrate faster than the original origami peak according to the separation principles of CE. However, it is unclear why the shifts in overall migration time and band broadening occur. Future experiments with incubation in different sample buffers can further elucidate the mechanism of DNA origami aggregation. Even so, these results demonstrate the potential for CE to detect morphological changes to DNA origami over time.

**Figure 7:**
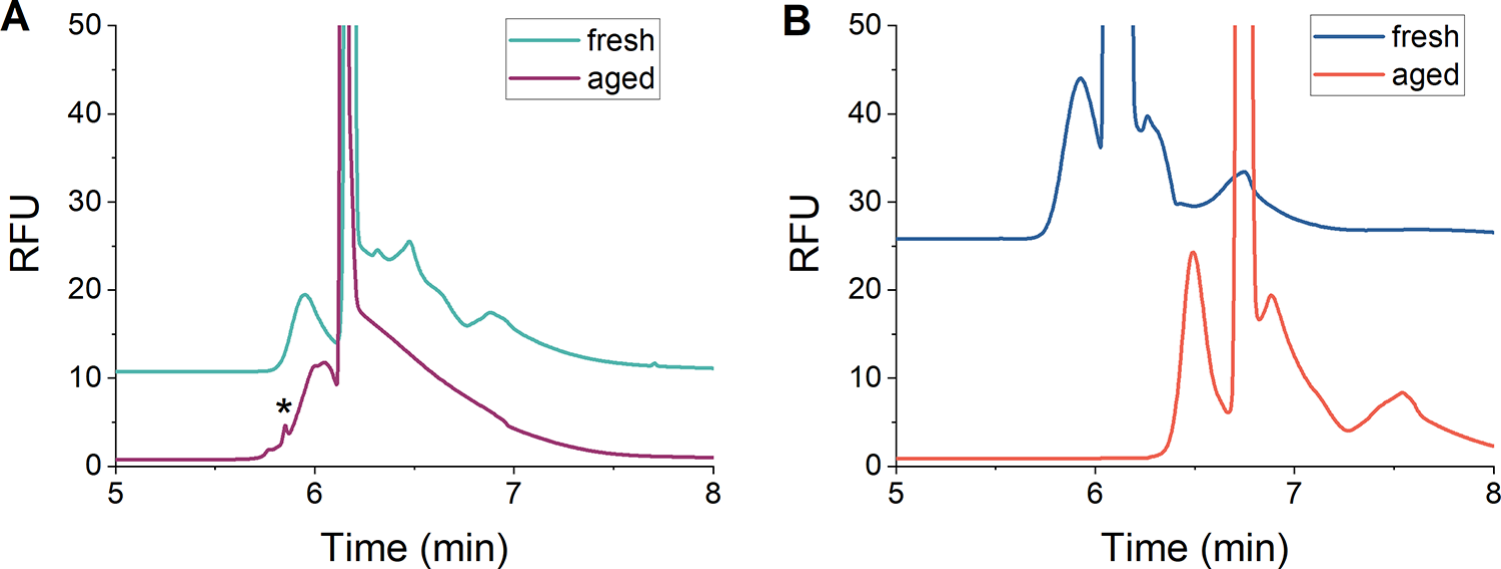
*ct*ITP electropherograms showing the separation of unpurified NR origami with (A) “no sides” or (B) “with sides”. Electropherograms were recorded immediately after dilution of the origami in the sample buffer (fresh) or after 1 week of incubation (aged). The appearance of an aggregate peak for the “no sides” sample is indicated with an asterisk (*). Electropherograms are vertically offset for clarity and the separation conditions were as indicated in Figure 3.

To confirm whether the new peak we observed was due to aggregation, we designed a NR sample with edge staples added (NR with sides, Figure S14B) to compare with the NR sample with no sides on the ends (NR no sides, Figure S14A). The NR sample with sides is more resistant to aggregation as it has staple strands that prevent base pairing between origami. A similar incubation experiment was performed to observe the potential difference between the two samples. The dye/buffer ratio was also increased to 1:25,000 in order to enhance the possible detection of aggregates. Small deviations in the electropherogram profile were observed, where the NR with no sides formed a non-Gaussian peak after 1 week and a new peak was observed at an earlier migration time (Figure 7A). Meanwhile, the NR with sides maintained a Gaussian peak; no new peaks were observed, and we only detected a shift to a later migration time even after 1 week of incubation (Figure 7B). As such, these differences demonstrate that the two structures do not have the same stability in the sample buffers, supporting the hypothesis that aggregation occurs in the NR sample with no sides and demonstrating that *ct*ITP has the necessary resolving power to detect aggregate structures.

## Conclusions

We described the systematic optimization and application of CZE and *ct*ITP for the analysis of DNA origami structures. *ct*ITP analysis was shown to have superior separation performance to CZE with regard to detection sensitivity (4-fold less dye used to label origami samples), resolution of analyte bands (*R*_s_ of 2.05 for *ct*ITP compared to 1.57 for CZE), peak symmetry (*A*_s_ of 1.29 for *ct*ITP compared to 0.213 for CZE), and peak efficiency (*N* of ≈12,000 for *ct*ITP compared to ≈1000 for CZE). We also systematically investigated the SYBR family of dyes (SYBR Gold, Green I, and Green II) for highly efficient on-column labeling of DNA origami samples and found SYBR Green I to offer the best balance of detection sensitivity and peak resolution. Finally, we demonstrated that the optimized *ct*ITP method can be applied to other origami structures (a tripod, rope, and pillar) and for the detection of aggregated origami structures.

Overall, CE-LIF shows substantial promise as a characterization tool for DNA origami samples, and with the potential for automated fraction collection,^21,22,32−34^ CE could have some utility for purification of small quantities of DNA origami. Given the distinct migration times observed for origami with different shapes, CE may also be used to detect misfolded origami structures. Additionally, if CE can be used to measure the aggregation of DNA origami, as our results suggest, it could be uniquely useful for characterizing the controlled aggregation used to create large monolayers of tiled DNA origami or the intermediate assemblies of larger 3D composite structures.^35,36^ Other applications have used DNA tiles to arrange proteins into different geometric patterns based on the properties of aptamer-directed assembly.^37^ Similarly, the immobilization of virus capsids, carbon nanotubes, and metal nanoparticles on DNA tiles has been studied for medical applications that require specific arrangements of biomolecules.^37−41^ In these cases, the high-resolving power of CE could be particularly useful to analyze the assemblies or confirm the immobilization of molecules and nanoparticles on the DNA origami structure. What’s more, the small sample volume requirements (nanoliter quantities) allow CE to be integrated into the DNA characterization workflow with minimal sample loss.

## Supporting Information Available

Supplementary figures and tables from CE optimization; AFM images of aggregated and nonaggregated NR structures (PDF)
Staple sequences to fold the origami with the M13MP18 scaffold (XLSX)

## References

[ref1] Rothemund P. W. K. Folding DNA to Create Nanoscale Shapes and Patterns. Nature 2006, 440 (7082), 297–302. 10.1038/nature04586. 16541064

[ref2] Nagamune T. Biomolecular Engineering for Nanobio/Bionanotechnology. Nano Convergence 2017, 4 (1), 9 10.1186/s40580-017-0103-4. 28491487 PMC5401866

[ref3] Xavier P. L.; Chandrasekaran A. R. DNA-Based Construction at the Nanoscale: Emerging Trends and Applications. Nanotechnology 2018, 29 (6), 062001 10.1088/1361-6528/aaa120. 29232197

[ref4] Wagenbauer K. F.; Engelhardt F. A. S.; Stahl E.; Hechtl V. K.; Stömmer P.; Seebacher F.; Meregalli L.; Ketterer P.; Gerling T.; Dietz H. How We Make DNA Origami. ChemBioChem 2017, 18 (19), 1873–1885. 10.1002/cbic.201700377. 28714559

[ref5] Scheible M.; Jungmann R.; Simmel F. C. Assembly and Microscopic Characterization of DNA Origami Structures. In Nano-Biotechnology for Biomedical and Diagnostic Research; Zahavy E.; Ordentlich A.; Yitzhaki S.; Shafferman A., Eds.; Springer Netherlands: Dordrecht, 2012; Vol. 733, pp 87–96. 10.1007/978-94-007-2555-3_9 22101715

[ref6] Thamm S.; Slesiona N.; Dathe A.; Csáki A.; Fritzsche W. AFM-Based Probing of the Flexibility and Surface Attachment of Immobilized DNA Origami. Langmuir 2018, 34 (49), 15093–15098. 10.1021/acs.langmuir.8b02362. 30252490

[ref7] Ramakrishnan S. Atomic Force Microscopy Studies of DNA Origami Nanostructures: From Structural Stability to Molecular Patterning, Dissertation; Universität Paderborn, 2018.

[ref8] LeBlanc S.; Wilkins H.; Li Z.; Kaur P.; Wang H.; Erie D. A. Using Atomic Force Microscopy to Characterize the Conformational Properties of Proteins and Protein–DNA Complexes That Carry Out DNA Repair. In Methods Enzymology; Elsevier, 2017; Vol. 592, pp 187–212. 10.1016/bs.mie.2017.04.004 PMC5761736 28668121

[ref9] Douglas S. M.; Dietz H.; Liedl T.; Högberg B.; Graf F.; Shih W. M. Self-Assembly of DNA into Nanoscale Three-Dimensional Shapes. Nature 2009, 459 (7245), 414–418. 10.1038/nature08016. 19458720 PMC2688462

[ref10] Liedl T.; Högberg B.; Tytell J.; Ingber D. E.; Shih W. M. Self-Assembly of Three-Dimensional Prestressed Tensegrity Structures from DNA. Nat. Nanotechnol. 2010, 5 (7), 520–524. 10.1038/nnano.2010.107. 20562873 PMC2898913

[ref11] Wei X.; Nangreave J.; Jiang S.; Yan H.; Liu Y. Mapping the Thermal Behavior of DNA Origami Nanostructures. J. Am. Chem. Soc. 2013, 135 (16), 6165–6176. 10.1021/ja4000728. 23537246

[ref12] Mathur D.; Medintz I. L. Analyzing DNA Nanotechnology: A Call to Arms For The Analytical Chemistry Community. Anal. Chem. 2017, 89 (5), 2646–2663. 10.1021/acs.analchem.6b04033. 28207239

[ref13] Heller C. Principles of DNA Separation with Capillary Electrophoresis. Electrophoresis 2001, 22 (4), 629–643. 10.1002/1522-2683(200102)22:4<629::AID-ELPS629>3.0.CO;2-S. 11296917

[ref14] Durney B. C.; Crihfield C. L.; Holland L. A. Capillary Electrophoresis Applied to DNA: Determining and Harnessing Sequence and Structure to Advance Bioanalyses (2009–2014). Anal. Bioanal. Chem. 2015, 407 (23), 6923–6938. 10.1007/s00216-015-8703-5. 25935677 PMC4551542

[ref15] McCord B.; Hartzell-Baguley B.; King S. Separation of DNA by Capillary Electrophoresis. In Capillary Electrophoresis; Schmitt-Kopplin P., Ed.; Humana Press: Totowa, NJ, 2008; pp 415–429. 10.1007/978-1-59745-376-9_15 18392577

[ref16] Smith A.; Nelson R. J. Capillary Electrophoresis of DNA. In Current Protocols in Nucleic Acid Chemistry; Beaucage S. L.; Bergstrom D. E.; Herdewijn P.; Matsuda A., Eds.; John Wiley & Sons, Inc.: Hoboken, NJ, 2003; pp 10.9.1–10.9.16. 10.1002/0471142700.nc1009s13 18428903

[ref17] Schiffels D.; Szalai V. A.; Liddle J. A. Molecular Precision at Micrometer Length Scales: Hierarchical Assembly of DNA–Protein Nanostructures. ACS Nano 2017, 11 (7), 6623–6629. 10.1021/acsnano.7b00320. 28651051 PMC11314666

[ref18] Majikes J. M.; Liddle J. A. DNA Origami Design: A How-To Tutorial. J. Res. Natl. Inst. Stand. 2021, 126, 126001 10.6028/jres.126.001. PMC11419732 39359737

[ref19] Hirokawa T.; Okamoto H.; Ikuta N.; Gas B. Optimization of Operational Modes for Transient Isotachophoresis Preconcentration-CZE. Anal. Sci. 2001, 17, i185–i188.

[ref20] Křivánková L.; Pantůčková P.; Boček P. Isotachophoresis in Zone Electrophoresis. J. Chromatogr. A 1999, 838 (1), 55–70. 10.1016/S0021-9673(99)00169-7. 10327640

[ref21] Saito S.; Hirose K.; Tsuchida M.; Wakui K.; Yoshimoto K.; Nishiyama Y.; Shibukawa M. Rapid Acquisition of High-Affinity DNA Aptamer Motifs Recognizing Microbial Cell Surfaces Using Polymer-Enhanced Capillary Transient Isotachophoresis. Chem. Commun. 2016, 52 (3), 461–464. 10.1039/C5CC07268A. 26525483

[ref22] Riley K. R.; Saito S.; Gagliano J.; Colyer C. L. Facilitating Aptamer Selection and Collection by Capillary Transient Isotachophoresis with Laser-Induced Fluorescence Detection. J. Chromatogr. A 2014, 1368, 183–189. 10.1016/j.chroma.2014.09.062. 25311485

[ref23] DeJaco R. F.; Majikes J. M.; Liddle J. A.; Kearsley A. J. Binding, Brightness, or Noise? Extracting Temperature-Dependent Properties of Dye Bound to DNA. Biophys. J. 2023, 122 (7), 1364–1375. 10.1016/j.bpj.2023.03.002. 36871160 PMC10111365

[ref24] Tuma R. S.; Beaudet M. P.; Jin X.; Jones L. J.; Cheung C.-Y.; Yue S.; Singer V. L. Characterization of SYBR Gold Nucleic Acid Gel Stain: A Dye Optimized for Use with 300-Nm Ultraviolet Transilluminators. Anal. Biochem. 1999, 268 (2), 278–288. 10.1006/abio.1998.3067. 10075818

[ref25] Cosa G.; Focsaneanu K. S.; McLean J. R.; McNamee J. P.; Scaiano J. C. Photophysical Properties of Fluorescent DNA-Dyes Bound to Single- and Double-Stranded DNA in Aqueous Buffered Solution. Photochem. Photobiol. 2001, 73 (6), 585–599. 10.1562/0031-8655(2001)073<0585:PPOFDD>2.0.CO;2. 11421063

[ref26] Zipper H.; Brunner H.; Bernhagen J.; Vitzthum F. Investigations on DNA Intercalation and Surface Binding by SYBR Green I, Its Structure Determination and Methodological Implications. Nucleic Acids Res. 2004, 32 (12), e103 10.1093/nar/gnh101. 15249599 PMC484200

[ref27] Kolbeck P. J.; Vanderlinden W.; Gemmecker G.; Gebhardt C.; Lehmann M.; Lak A.; Nicolaus T.; Cordes T.; Lipfert J. Molecular Structure, DNA Binding Mode, Photophysical Properties and Recommendations for Use of SYBR Gold. Nucleic Acids Res. 2021, 49 (9), 5143–5158. 10.1093/nar/gkab265. 33905507 PMC8136779

[ref28] Silva G. L.; Ediz V.; Yaron D.; Armitage B. A. Experimental and Computational Investigation of Unsymmetrical Cyanine Dyes: Understanding Torsionally Responsive Fluorogenic Dyes. J. Am. Chem. Soc. 2007, 129 (17), 5710–5718. 10.1021/ja070025z. 17411048 PMC2535610

[ref29] Zhang X.; McGown L. B. Sequence-Based Separation of Single-Stranded DNA Using Nucleotides in Capillary Electrophoresis: Focus on Phosphate: CE and CEC. Electrophoresis 2013, 34 (12), 1778–1786. 10.1002/elps.201200683. 23576075 PMC3906628

[ref30] Nowak P. M.; Woźniakiewicz M.; Gładysz M.; Janus M.; Kościelniak P. Improving Repeatability of Capillary Electrophoresis—a Critical Comparison of Ten Different Capillary Inner Surfaces and Three Criteria of Peak Identification. Anal. Bioanal. Chem. 2017, 409 (18), 4383–4393. 10.1007/s00216-017-0382-y. 28484810 PMC5486911

[ref31] Fritsch R. J.; Krause I. Electrophoresis. In Encyclopedia of Food Sciences and Nutrition; Elsevier, 2003; pp 2055–2062.

[ref32] Riley K. R.; Gagliano J.; Xiao J.; Libby K.; Saito S.; Yu G.; Cubicciotti R.; Macosko J.; Colyer C. L.; Guthold M.; Bonin K. Combining Capillary Electrophoresis and Next-Generation Sequencing for Aptamer Selection. Anal. Bioanal. Chem. 2015, 407 (6), 1527–1532. 10.1007/s00216-014-8427-y. 25579462 PMC4329186

[ref33] Stuart C. H.; Riley K. R.; Boyacioglu O.; Herpai D. M.; Debinski W.; Qasem S.; Marini F. C.; Colyer C. L.; Gmeiner W. H. Selection of a Novel Aptamer Against Vitronectin Using Capillary Electrophoresis and Next Generation Sequencing. Mol. Ther. Nucleic Acids 2016, 5 (11), e386 10.1038/mtna.2016.91. 27845768 PMC5155323

[ref34] Haraga T.; Tsujimura H.; Miyauchi S.; Kamimura T.; Shibukawa M.; Saito S. Purification of Anionic Fluorescent Probes through Precise Fraction Collection with a Two-Point Detection System Using Multiple-Stacking Preparative Capillary Transient Isotachophoresis. Electrophoresis 2020, 41 (13–14), 1152–1159. 10.1002/elps.201900399. 32253765

[ref35] Li Z.; Liu M.; Wang L.; Nangreave J.; Yan H.; Liu Y. Molecular Behavior of DNA Origami in Higher-Order Self-Assembly. J. Am. Chem. Soc. 2010, 132 (38), 13545–13552. 10.1021/ja106292x. 20825190 PMC3071357

[ref36] Wagenbauer K. F.; Sigl C.; Dietz H. Gigadalton-Scale Shape-Programmable DNA Assemblies. Nature 2017, 552 (7683), 78–83. 10.1038/nature24651. 29219966

[ref37] Chhabra R.; Sharma J.; Ke Y.; Liu Y.; Rinker S.; Lindsay S.; Yan H. Spatially Addressable Multiprotein Nanoarrays Templated by Aptamer-Tagged DNA Nanoarchitectures. J. Am. Chem. Soc. 2007, 129 (34), 10304–10305. 10.1021/ja072410u. 17676841

[ref38] Stephanopoulos N.; Liu M.; Tong G. J.; Li Z.; Liu Y.; Yan H.; Francis M. B. Immobilization and One-Dimensional Arrangement of Virus Capsids with Nanoscale Precision Using DNA Origami. Nano Lett. 2010, 10 (7), 2714–2720. 10.1021/nl1018468. 20575574 PMC3083853

[ref39] Shen X.; Asenjo-Garcia A.; Liu Q.; Jiang Q.; García de Abajo F. J.; Liu N.; Ding B. Three-Dimensional Plasmonic Chiral Tetramers Assembled by DNA Origami. Nano Lett. 2013, 13 (5), 2128–2133. 10.1021/nl400538y. 23600476

[ref40] Carbone A.; Seeman N. C. Circuits and Programmable Self-Assembling DNA Structures. Proc. Natl. Acad. Sci. U.S.A. 2002, 99 (20), 12577–12582. 10.1073/pnas.202418299. 12232051 PMC130502

[ref41] Woods D.; Doty D.; Myhrvold C.; Hui J.; Zhou F.; Yin P.; Winfree E. Diverse and Robust Molecular Algorithms Using Reprogrammable DNA Self-Assembly. Nature 2019, 567 (7748), 366–372. 10.1038/s41586-019-1014-9. 30894725

