# Supplementary material for: Analysis of DNA Origami Nanostructures Using Capillary Electrophoresis
Source: Anal Chem. 2023 Dec 13;95(51):18783–92. doi: 10.1021/acs.analchem.3c03641 (PMC10753524; doi:10.1021/acs.analchem.3c03641)
Supplement: Supplementary file 1 — ac3c03641_si_001.pdf [file ac3c03641_si_001.pdf]

Supporting Information:

Analysis of DNA Origami Nanostructures Using Capillary Electrophoresis

Janan Hui[1], Jacob M. Majikes[2], Kathryn R. Riley[1,*]

[1]Department of Chemistry and Biochemistry, Swarthmore College, Swarthmore, PA 19081, USA
[2] Physical Measurement Laboratory, National Institute of Standards and Technology (NIST), Gaithersburg, MD 20899, USA

*To whom correspondence should be addressed; email: kriley1@swarthmore.edu tel: +1-610-328-7369

Contents

Equations S1-S3. Calculation of Analytical Figures of Merit

Figure S1. Peak identification of NR using CZE

Figure S2. Pre- vs. on-column labeling of DNA origami using *ct*ITP

Table S1. Optimized CZE separation conditions

Table S2. Optimization of CZE separation parameters

Figure S3. CZE optimization of dye concentration

Figure S4. CZE optimization of buffer pH

Figure S5. CZE optimization of Mg^{2+} concentration

Figure S6. CZE optimization of Tris concentration

Figure S7. CZE optimization of buffer composition

Figure S8. CZE optimization of capillary length

Figure S9. *ct*ITP optimization of injection volume

Table S3. Optimization of *ct*ITP separation parameters

Figure S10. *ct*ITP optimization of Tris concentration

Figure S11. *ct*ITP optimization of glycine concentration

Table S4. Optimized CZE and *ct*ITP separation conditions

Figure S12. Gel electrophoresis of filtered and unfiltered DNA origami samples

Figure S13. Identification of NR aggregates using *ct*ITP

Figure S14. AFM images of NR with and without sides

Equations S1-S3. Calculation of Analytical Figures of Merit.

The resolution factor, R_S, was used to quantify the effectiveness of the optimized separation according to:

$$R_s = \frac{t_1 - t_2}{\frac{1}{2}(W_1 - W_2)} \qquad \textbf{(S1)}$$

where t_1 and t_2 represent the migration time of each peak and W_1 and W_2 represent the width of each peak.

The number of theoretical plates, *N*, was calculated to quantify the peak efficiency for each separation mode according to:

$$N = 16\left(\frac{t_R}{W}\right)^2 \qquad \textbf{(S2)}$$

where t_R represents the retention time of the NR origami on the column, and W represents the width of the origami peak.

The peak asymmetry factor, A_S, was also calculated to show the improvement in the Gaussian nature of each peak, according to:

$$A_s = \frac{b}{a} \qquad \textbf{(S3)}$$

where b represents the distance from the leading point of the peak to the maximum point at 20 % of the peak height, and a represents the distance from the trailing point of the peak to the maximum point at 20 % of the peak height. An $A_s > 1$ indicates tailing and $A_s < 1$ indicates fronting.

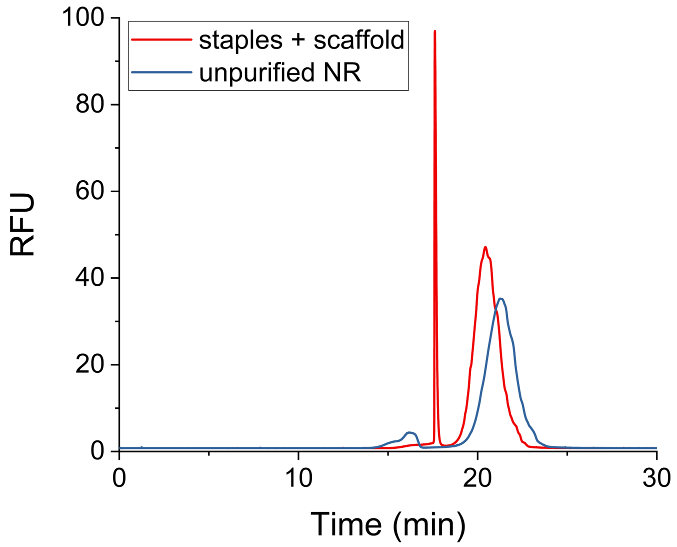

Figure S1. CZE electropherograms of a mixture of DNA staples and the scaffold compared to unpurified NR. Samples were prepared in 40 mmol.L^{-1} Tris, 12.5 mmol.L^{-1} $Mg(CH_3COO)_2$, 1 mmol.L^{-1} EDTA, pH 7.5 with 1:25,000 SYBR Green I: buffer volume ratio.

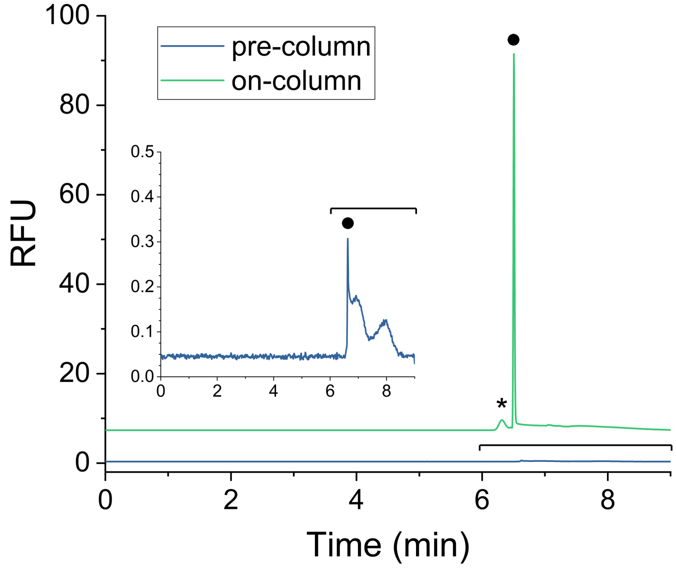

Figure S2. NR DNA origami sample was injected using optimized *ct*ITP conditions with on-column (green) and pre-column (blue) labeling with SYBR Green I dye to show the 300× difference in fluorescent signal. The electropherogram of the pre-column labeled NR sample is magnified to highlight the existence of the staples peak in the bracketed area (left, inset), and the on-column electropherogram is vertically offset by 10 RFU for clarity. The NR origami peak is marked with an asterisk and the staples peak is marked with a circle.

Table S1. Optimized CZE separation conditions

System	Separation Voltage (kV)	Injection time (psi*s)	L_{total}, $L_{effective}$ (cm)	Capillary I.D. (μm)	Dye: buffer	pH	[Tris] (mM)	[Mg^{2+}] (mM)	[EDTA] (mM)
Initial	20	4*5	40.2, 30.0	50	1:100,000	7.5	40	12.5	1.0
Final	20	4*5	60.2, 50.0	50	1:25,000	8.0	60	5.0	1.0

Table S2. Optimization of CZE separation parameters as measured by the %RSD of the DNA staples peak migration time, area, and width[a]

**Separation Parameter**	**Evaluated Range**[b]	**%RSD**		
		**Migration Time**	**Peak Area**	**Peak Width**
[SYBR Gold] (as a ratio of the 10,000× concentrate)	1:100,000	5.6	6.3	16.7
	1:50,000	5.7	4.9	3.3
	**1:25,000**	**1.9**	**1.2**	**3.3**
	1:10,000	2.6	7.1	17.0
buffer pH	7.0	12.4	29.3	16.2
	7.5	1.0	9.2	4.2
	**8.0**	**7.9**	**2.6**	**10.6**
	8.5	2.1	16.9	2.3
	9.0	28.2	88.8	70.8
[Mg^{2+}] ($mmol.L^{-1}$)	2.5	0.9	23.1	6.3
	**5.0**	**1.4**	**2.9**	**5.4**
	7.5	1.1	32.9	1.2
	10.0	1.0	6.5	3.8
	12.5	1.7	3.5	2.7
[Tris] ($mmol.L^{-1}$)	10	26.1	48.6	34.4
	20	16.9	8.2	15.6
	30	8.4	9.2	21.2
	40	4.6	17.4	7.1
	**60**	**0.7**	**0.1**	**2.7**
Buffer composition (all 60 $mmol.L^{-1}$)	MOPS	1.7	5.3	5.1
	HEPES	3.7	15.9	8.2

[a]For each parameter, the chosen conditions are in bold. The row highlighted in blue represents the final chosen conditions.
[b]As each separation parameter was optimized, the other parameters were kept constant according to Figures S3-S7.

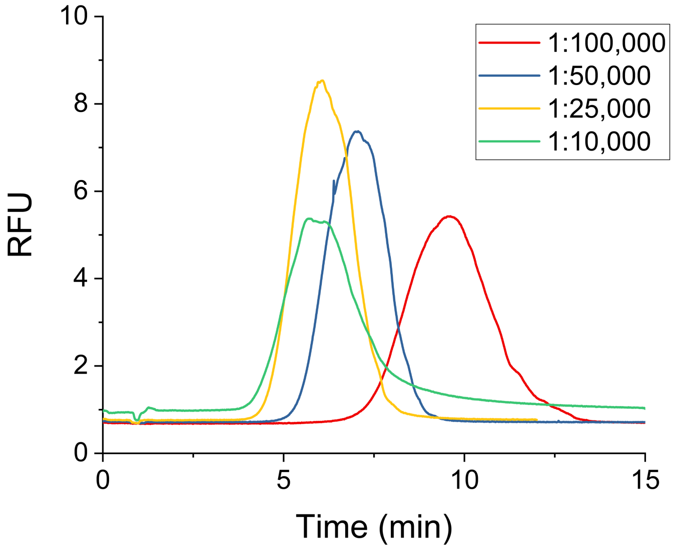

Figure S3. CZE electropherograms of DNA staples prepared in 40 mmol.L^{-1} Tris, 12.5 mmol.L^{-1} $Mg(CH_3COO)_2$, 1 mmol.L^{-1} EDTA, pH 7.5 with varying dye concentration (indicated in the legend as the SYBR Gold: buffer volume ratio).

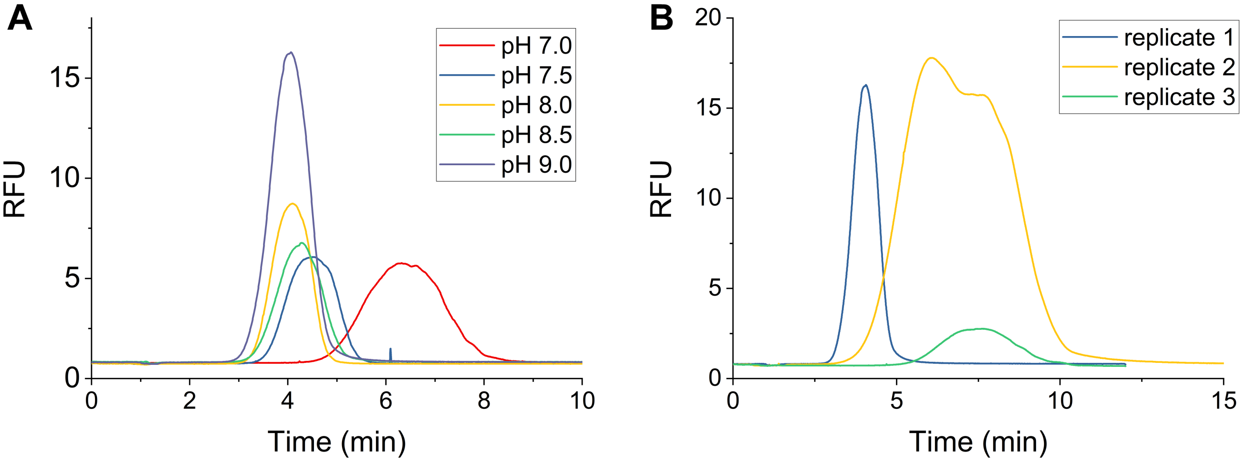

Figure S4. (A) Effect of buffer pH on CZE electropherograms of DNA staples prepared in 40 mmol.L^{-1} Tris, 12.5 mmol.L^{-1} $Mg(CH_3COO)_2$, 1 mmol.L^{-1} EDTA, 1:25,000 SYBR Gold: buffer ratio, with the pH indicated in the legend. (B) Replicate CZE electropherograms of DNA staples showing the irreproducible peak profile. Staples were prepared in 40 mmol.L^{-1} Tris, 12.5 mM mmol.L^{-1} $MgCl_2$, 1 mmol.L^{-1} EDTA, 1:25,000 dye: buffer ratio, pH 9.0.

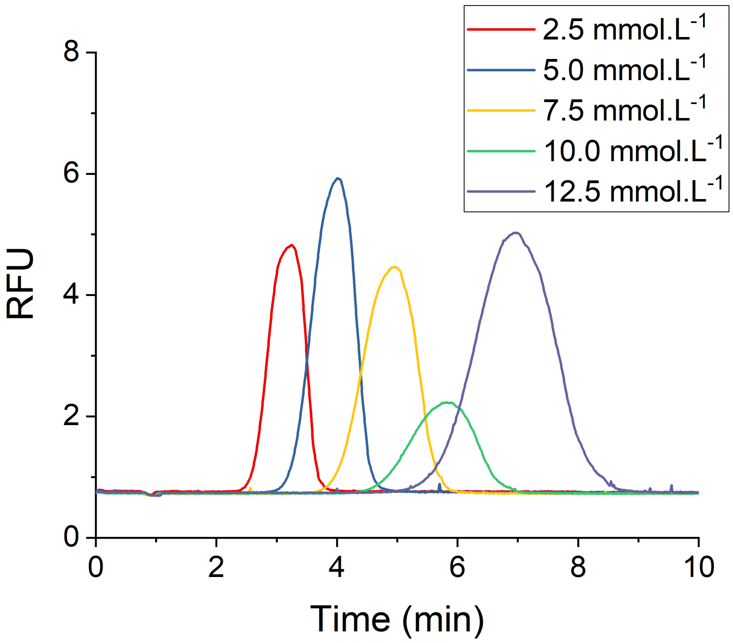

Figure S5. Electropherograms of DNA staples in 40 mmol.L^{-1} Tris, 1 mmol.L^{-1} EDTA, 1:25,000 dye: buffer ratio, pH 8.0, and varying concentration of $Mg(CH_3COO)_2$ as indicated in the legend.

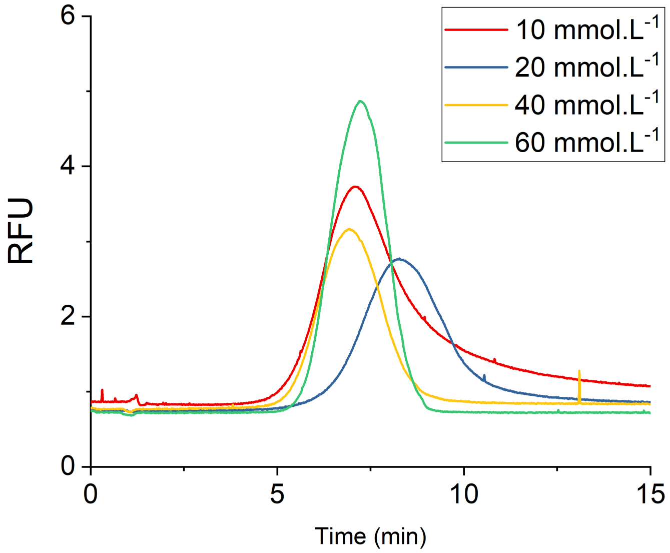

Figure S6. Effect of ionic strength on CZE electropherograms of DNA staples prepared in 5.0 mmol.L^{-1} $Mg(CH_3COO)_2$, 1 mmol.L^{-1} EDTA, 1:25,000 dye: buffer ratio, pH 8.0, with Tris concentration indicated in the legend.

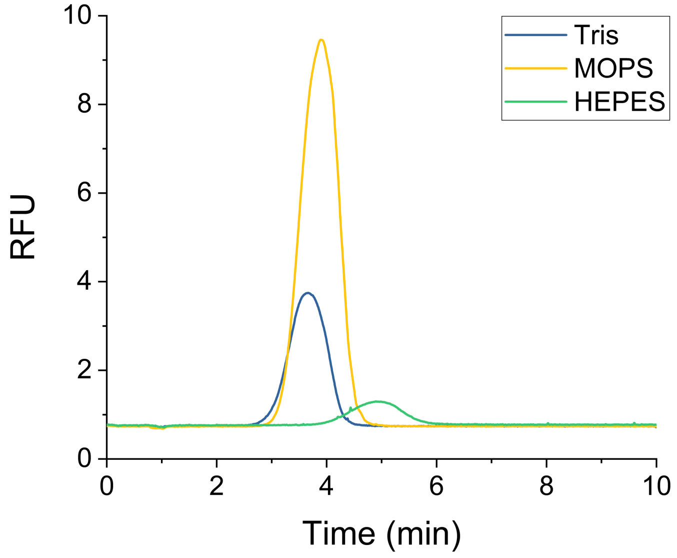

Figure S7. Effect of zwitterionic buffer type on electropherograms of staples sample prepared in 5.0 mmol.L^{-1} $Mg(CH_3COO)_2$, 1 mmol.L^{-1} EDTA, 1:25,000 dye: buffer ratio, pH 8.0, and 60 mmol.L^{-1} Tris, MOPS or HEPES.

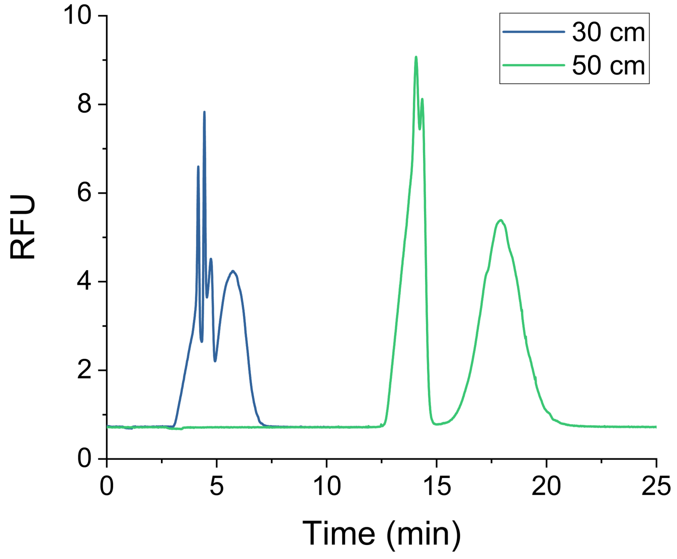

Figure S8. Electropherograms of scaffold and staples prepared in 60 mmol.L^{-1} Tris, 5.0 mmol.L^{-1} $Mg(CH_3COO)_2$, 1 mmol.L^{-1} EDTA, 1:25,000 dye: buffer ratio, and pH 8.0 and separated on capillaries with different effective lengths as indicated in the legend.

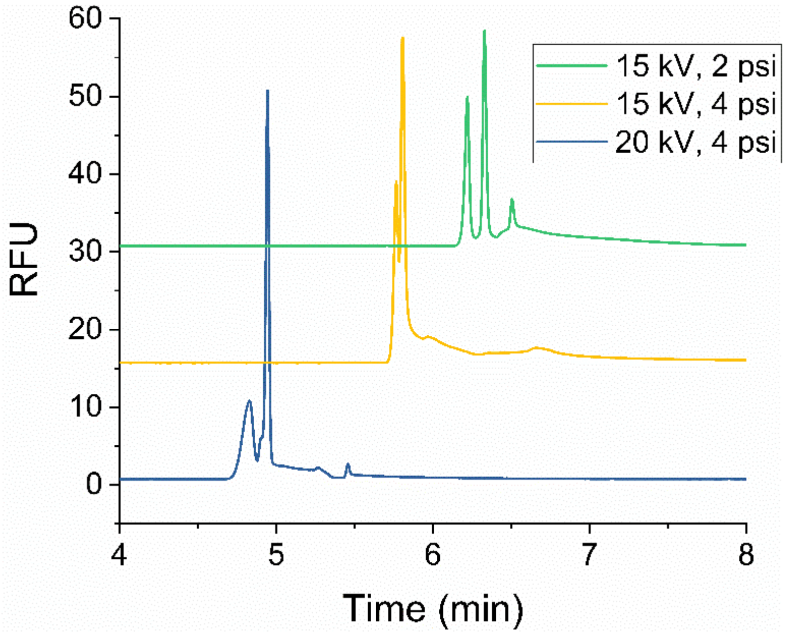

Figure S9. Effect of injection conditions on *ct*ITP electropherograms of NR-staples sample mix in optimized sample buffer and with separation buffer of 31 mmol.L^{-1} Tris, 500 mM glycine, 1:100,000 dye: buffer ratio, with injection parameters indicated by the legend. Electropherograms are offset at 15 RFU increments for clarity and all other separation conditions are as reported in Table S4.

Table S3. Optimization of *ct*ITP separation parameters as measured by the resolution factor, R_s, between the NR and DNA staples[a]

**Separation Parameter**	**Evaluated Range**[b]	$\boldsymbol{R_s}$
Injection Volume (nL)	**19**	**0.36 ± 0.06**
	38	0.04 ± 0.01
Separation Voltage (kV)	20	0.04 ± 0.01
	**15**	**0.17 ± 0.05**
[Tris] (mmol.L^{-1})	10	0.13 ± 0.06
	20	0.45 ± 0.15
	31	0.12 ± 0.02
	**40**	**0.50 ± 0.02**
	50	0.37 ± 0.01
[Gly] (mmol.L^{-1})	100	0.06 ± 0.07
	200	0.09 ± 0.03
	300	0.17 ± 0.02
	400	0.23 ± 0.08
	**500**	**0.62 ± 0.15**
	600	0.43 ± 0.07
	700	0.29 ± 0.04
	800	0.08 ± 0.03
	900	0.13 ± 0.01
	1000	0.13 ± 0.2

[a]For each parameter, the chosen conditions are in bold.
[b]As each separation parameter was optimized, the other parameters were kept constant according to Figures S9-S11.

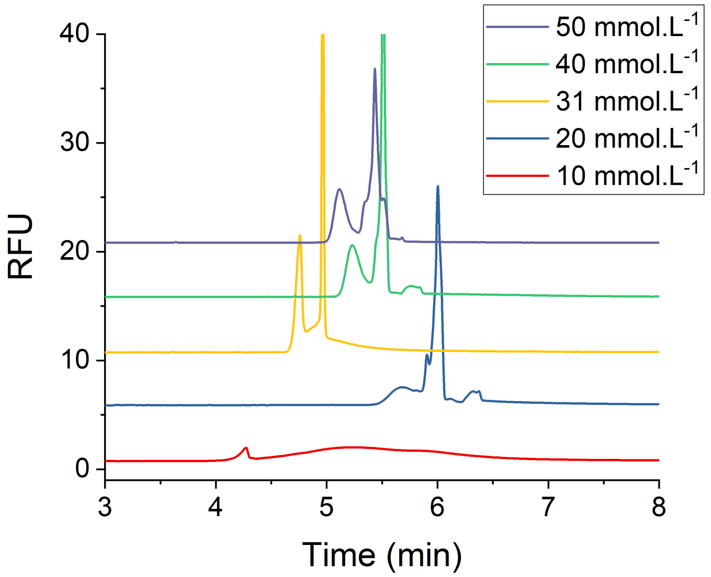

Figure S10. Effect of Tris concentration on *ct*ITP electropherograms of NR-staples sample mix in optimized sample buffer and with separation buffer of 500 mmol.L^{-1} Glycine, 1:100,000 dye: buffer ratio, with different Tris concentration indicated in the legend. Electropherograms are offset at 5 RFU increments for clarity and all other separation conditions are as reported in Table S4.

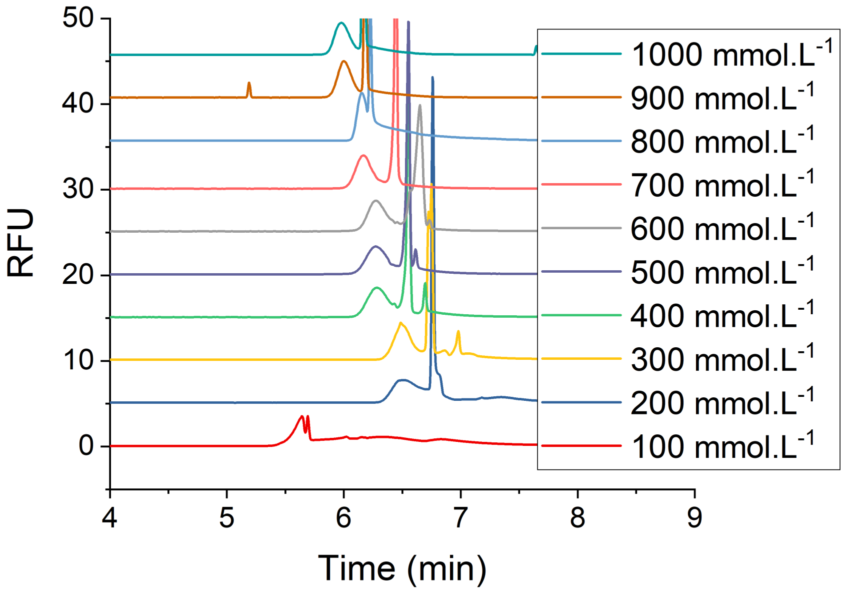

Figure S11. Effect of glycine concentration on electropherograms of NR-staples sample mix in optimized sample buffer and with separation buffer of 40 mmol.L^{-1} Tris, 1:100,000 dye: buffer ratio, with different glycine concentration indicated in the legend. Electropherograms are offset at 5 RFU increments for clarity and all other separation conditions are as reported in Table S4.

Table S4. Optimized CZE and *ct*ITP separation conditions

CE Mode	Separation Voltage (kV)	Injection time (psi*s)	L_{total}, $L_{effective}$ (cm)	Capillary I.D. (μm)	SYBR Green I: buffer	Buffer composition
CZE	20	4*5	60.2, 50	50	1:25,000	60 mM Tris, 5.0 mM Mg^{2+}, 1.0 mM EDTA, pH 8.0
*ct*ITP	15	2*5	60.2, 50	50	1:100,000	*Sample buffer:*<br>60 mM Tris, 5.0 mM Mg^{2+}, 1.0 mM EDTA, pH 8.0<br>*Separation buffer:*<br>40 mM Tris, 500 mM Gly, ≈pH 8.5

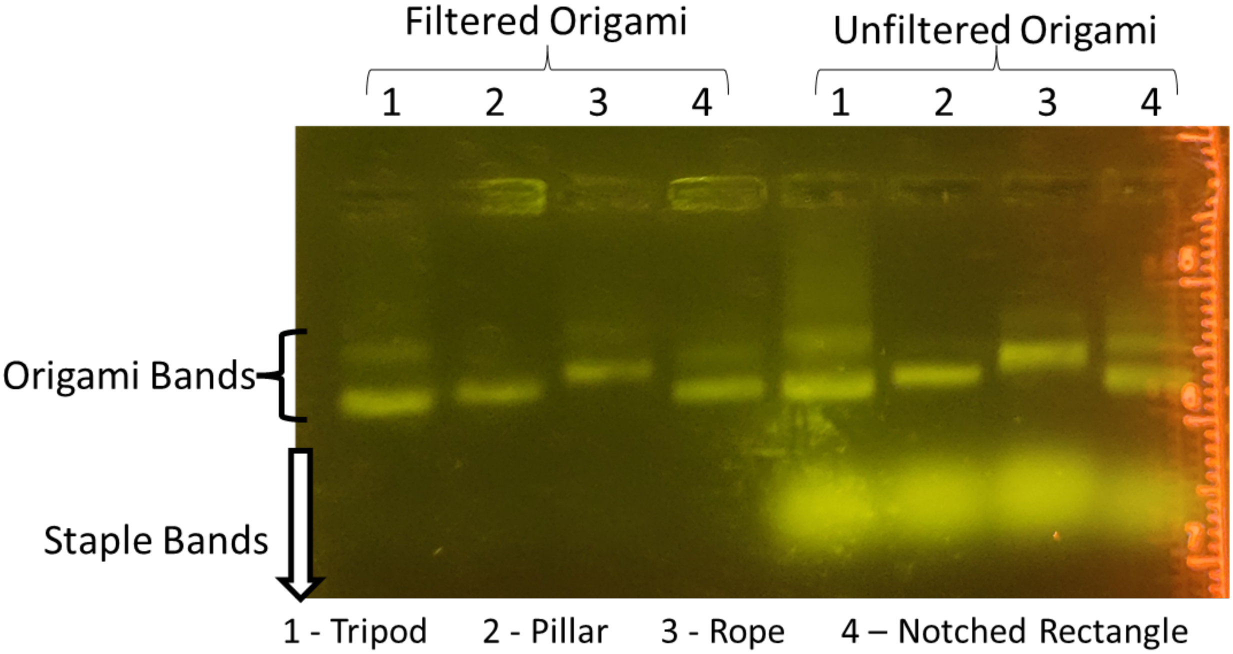

Figure S12. Gel electrophoresis of spin filtered and unfiltered DNA origami. Gel electrophoresis was performed in 1 % agarose gel in 1× TAE buffer supplemented with 12.5 mmol.L^{-1} Mg^{2+}, run for 30 minutes at 90 V (approximately 450 V.m^{-1}), and stained with Sybr Green II.

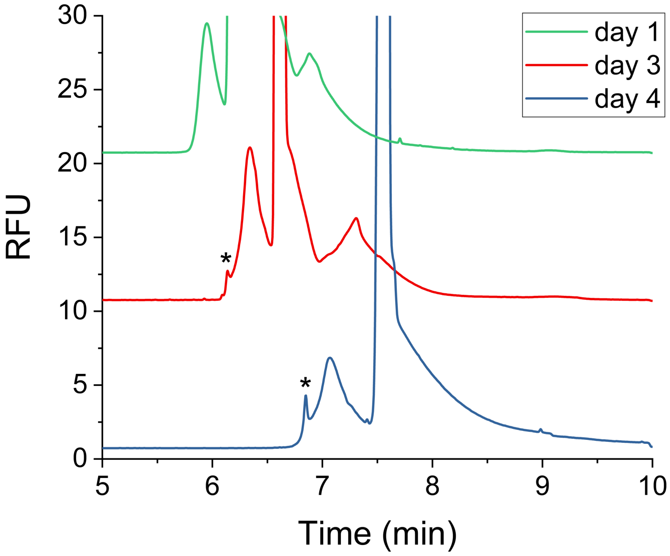

Figure S13. *ct*ITP electropherograms showing the NR sample without sides, freshly prepared (day 1) or after aging in buffer for 3 or 4 days. Separation conditions are as reported in Table S4.

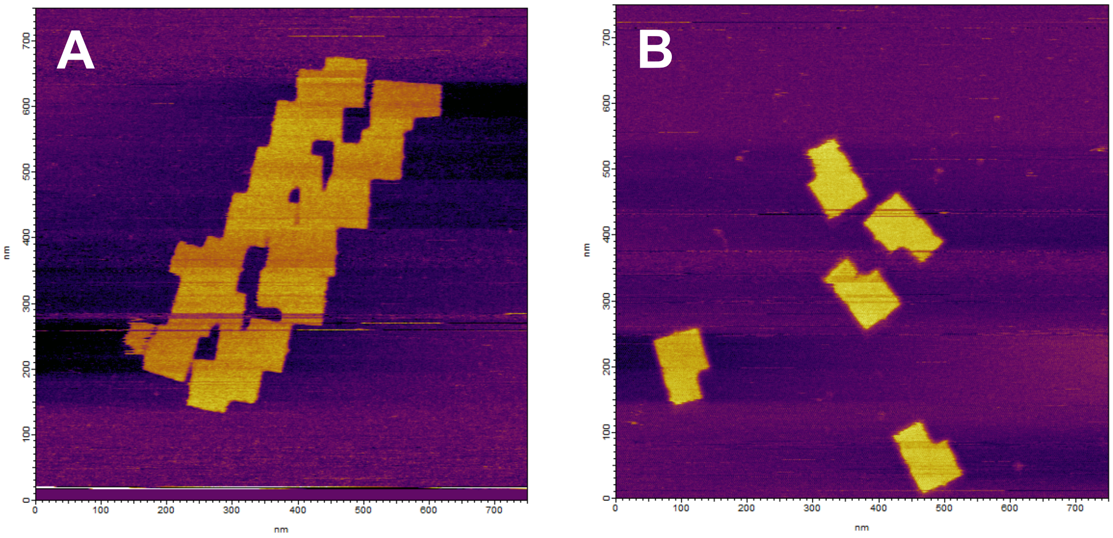

Figure S14. AFM micrographs comparing the NR **(A)** without sides or **(B)** with sides. The NR without sides is prone to aggregation.
